# Emerging roles of circular RNAs in nasopharyngeal carcinoma: functions and implications

**DOI:** 10.1038/s41420-024-01964-x

**Published:** 2024-04-25

**Authors:** Aiyu Ma, Yuzhong Yang, Lu Lu, Yan Zhang, Xuemei Zhang, Jinhua Zheng, Xiang Zheng

**Affiliations:** 1 Department of Pathology, Affiliated Hospital of Guilin Medical University, Guilin, Guangxi China; 2 Department of Pathology, Liuzhou People’s Hospital Affiliated to Guangxi Medical University, Liuzhou, Guangxi China

**Keywords:** Head and neck cancer, Tumour biomarkers

## Abstract

Nasopharyngeal carcinoma (NPC) is a distinct malignancy primarily prevalent in Southern China and Southeast Asia. Circular RNAs (circRNAs), a class of non-coding RNAs, are evolutionarily conserved and exhibit remarkable stability. Their dysregulation has been observed in various cancers, including NPC. In this review, we investigate the pivotal role of circRNAs in NPC, focusing specifically on their involvement in tumor proliferation, apoptosis, metastasis, angiogenesis, stemness, metabolism, and the tumor microenvironment. We highlight the diagnostic and prognostic potential of circRNAs in NPC, emphasizing their utility as biomarkers for early detection, disease monitoring, and prediction of treatment outcomes. Additionally, we explore the therapeutic implications of circRNAs in NPC, highlighting their potential for targeted therapies.

## FACTS

- CircRNAs are implicated in the pathogenesis of various diseases, including Alzheimer’s disease, osteoarthritis, atherosclerosis, and cancer.
- Research focusing on circRNAs in NPC has provided abundant evidence that circRNAs are involved in regulating the progression of NPC.
- Early diagnosis and intervention play a pivotal role in improving the prognosis of NPC, and circRNA has emerged as a promising biomarker for NPC diagnosis and prognosis.

## OPEN QUESTIONS

- How to utilize a circRNA-targeted individualized treatment strategy for NPC?
- Further investigation is required to explore the regulatory mechanism of circRNA-encoded peptides on the progression of NPC.
- The identification of additional circRNA biomarkers in NPC with definitive diagnostic and prognostic value is still needed.

## Introduction

NPC is a rare yet severe epithelial tumor characterized by significant geographical and demographic variations. It is particularly prevalent in Southern China and Southeast Asia [1, 2]. The causes of NPC are multifactorial, involving genetic factors, environmental influences, Epstein-Barr Virus (EBV) infection, oral hygiene, smoking, and other factors [3–6]. Notably, EBV plays a crucial role in the development of NPC, as its genome is nearly always present in the malignant cells of undifferentiated NPC [7]. Currently, radiotherapy is the primary treatment approach for NPC. Although early-stage NPC often exhibits a high cure rate, the prognosis for patients with advanced-stage can be highly variable, and achieving complete tumor eradication is not always feasible [8]. Therefore, early detection, timely diagnosis, and prompt intervention are of paramount importance for improving the prognosis of NPC.

CircRNAs, a type of non-coding RNA (ncRNA), are highly abundant, evolutionarily conserved, and known for their remarkable stability. In the past, circRNAs were believed to be produced as byproducts of erroneous splicing. However, with advancements in science and technology, circRNAs have been found to have multiple biological functions [9, 10]. Numerous studies have demonstrated the significant role of circRNAs in the occurrence and development of various cancers. For example, *circPDIA4* is identified as an oncogenic circRNA in gastric cancer, exerting its oncogenic functions through distinct mechanisms in both the cytoplasm and the nucleus [11]. In bladder cancer, *circPTK2* enhances the stability of *SETDB1* mRNA by binding to PABPC1, subsequently facilitating SETDB1 expression. *CircPTK2*/PABPC1/SETDB1 pathway promotes metastasis and gemcitabine resistance of bladder cancer [12]. Extensive research also highlights the significant role of circRNAs in NPC pathogenesis and progression, such as *circRILPL1*, which activates the Hippo-YAP signaling pathway by interacting with ROCK1 and IPO7, thereby promoting NPC proliferation and metastasis [13]. These studies provide valuable insights into the molecular mechanisms of circRNAs in cancer and potential therapeutic targets. In this review, we provide an overview of the principles governing circRNA formation and highlight their influence on the initiation and progression of NPC. Additionally, we explore the potential value of circRNAs as diagnostic, prognostic markers as well as therapeutic targets in NPC.

## Biosynthesis and degradation of circRNAs

CircRNAs were first observed in the 1970s, with their initial discovery in plant pathogens known as viroids [14, 15]. For many years after their discovery, circRNAs were primarily regarded as molecular oddities or byproducts of splicing errors rather than functional molecules [16]. However, the view on circRNAs has started to change with advancement of RNA sequencing technologies and bioinformatics. In the 21st century, the development of these technologies has enabled researchers to study circRNAs more comprehensively [17, 18]. As a result, the potential roles of circRNAs in various biological processes are beginning to emerge. This change in understanding has sparked a growing interest in circRNAs and their functional significance in biology.

**Fig. 1:**
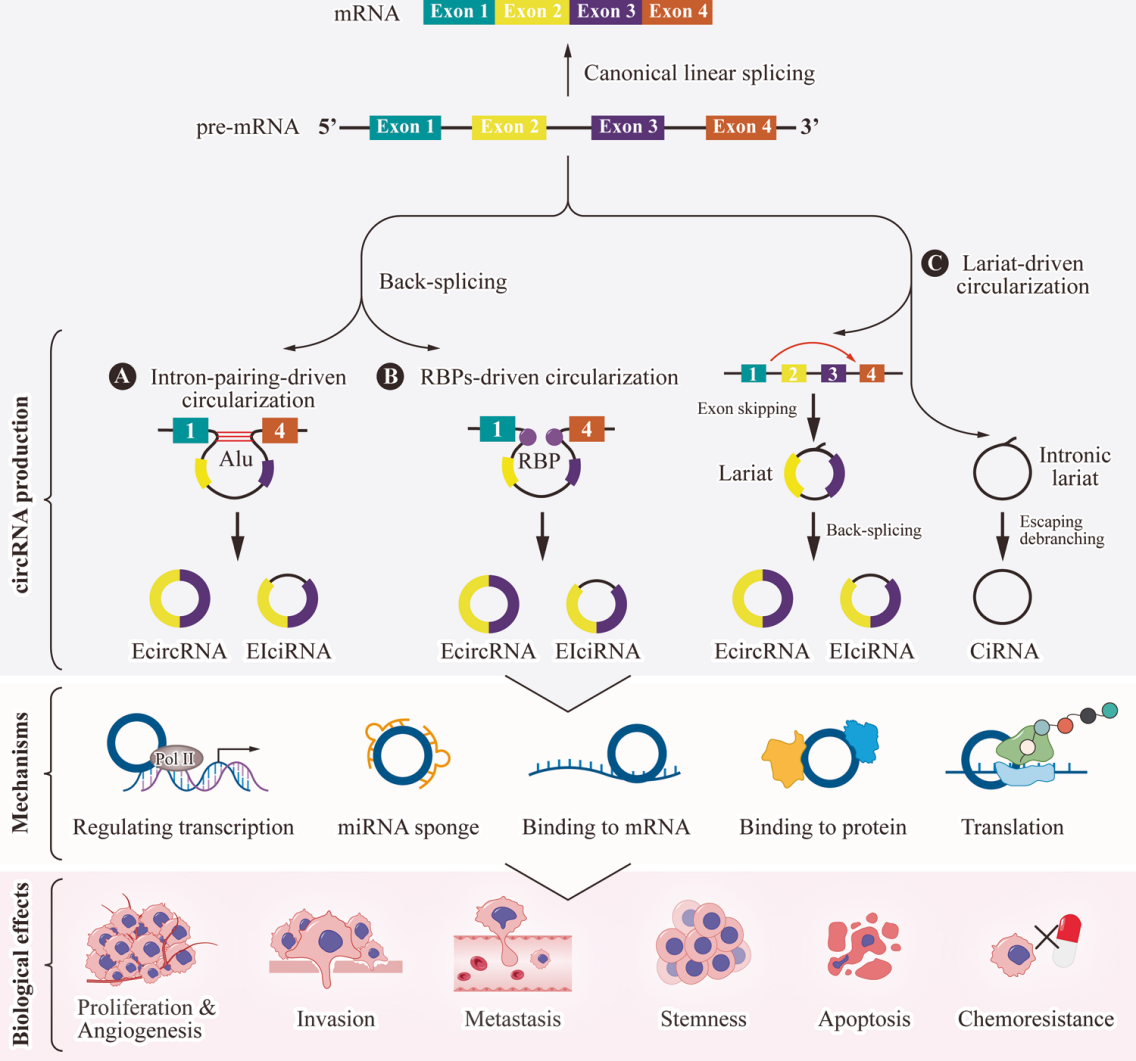
The production models, mechanisms, and biological effects of circRNAs. **A** Flanking inverted repeat elements (such as Alu elements) and **B** trans-acting RBPs favor back-splicing, resulting in the formation of EcircRNAs or EIciRNAs. **C** Exon-skipping events during splicing can generate a lariat structure, facilitating circularization, resulting in the formation of EcircRNAs or EIciRNAs. CiRNAs generate from intronic lariat precursors that escape the debranching step. After production, circRNAs can function as miRNA sponges, interact with proteins and mRNA, translate into functional peptides. Additionally, some circRNAs may interact with the RNA polymerase II (Pol II) complex in the promoter region of targeted genes, regulating the transcription of genes. These mechanisms contribute to their various biological effects in regulating tumor progression, such as proliferation, invasion, metastasis, angiogenesis, stemness, apoptosis, chemotherapy resistance, and so on.

CircRNAs are typically classified into three main types: exonic circRNAs (EcircRNAs), circular intronic circRNAs (CiRNAs), and exon-intron circRNAs (EIciRNAs) [19, 20]. EcircRNAs consist solely of exonic material, forming through back-splicing events where the 3’ end of one exon joins with the 5’ end of an upstream exon. CiRNAs originate from intronic sequences and are relatively rare. They are created from lariat introns that evade debranching [21]. EIciRNAs contain both exonic and intronic sequences and are thought to regulate the expression of their parent genes. CircRNAs possess unique characteristics due to their distinctive structure. Unlike linear RNAs, circRNAs adopt a covalently closed continuous loop structure, lacking both 5’ to 3’ ends and polyadenylated tails [22]. This circular configuration significantly enhances their stability, making them resistant to degradation by exonucleases, ensuring their robust stability and accumulation within cells [10]. In contrast to the canonical splicing that connects upstream splicing donor sites to downstream splicing acceptor sites, many circRNAs are formed through back-splicing, where downstream splicing donor sites are joined to upstream splicing acceptor sites in the opposite direction [23–25]. There are at least three models for circRNA production (Fig. 1). First model is driven by pairing in introns near the 5’ splice sites and branch points, which is referred to as intron-pairing-driven circularization [26]. This model can be catalyzed either by complementary base pairing or by inverted repeated ALU complementary flanking elements on introns [18, 25]. Another model involved in circRNA formation is mediated by trans-acting elements, specifically RNA binding proteins (RBPs), which is referred to as RBP-driven circularization. Several RBPs are found to have the capability to bind to pre-mRNAs, facilitating the connection between donor and recipient sequences and thereby promoting circRNAs formation. For example, muscleblind (MBL/MBNL1) protein can bind to its own pre-mRNA flanking introns of circularized exons [25]. This binding is thought to bring the splice sites closer together, promoting back-splicing and the formation of circRNAs. FUS protein binds with the 3′ start flanking intron region of *pre-ROBO1* to facilitate the back-splicing event leading to the formation of *circROBO1* [27]. CircRNAs can be also generated through the lariat-driven circularization. Exon-skipping event during splicing leads to the formation of a lariat whose restricted structure promotes circularization. When the lariat containing skipped exons undergoes back-splicing, it results in the formation of circRNAs. Additionally, ciRNAs can also be derived from intronic lariat precursors that successfully evade the debranching step [28, 29].

Multiple pathways are involved in the degradation of circRNA. Notably, RBPs also participate in circRNA degradation. One such RBP is adenosine deaminase acting on RNA 1 (ADAR1) that can suppress the formation of circRNAs. ADAR1 binds to double-stranded RNA (dsRNA) structures formed by base-pairing within flanking introns of circularized exons. Through ADAR1-mediated A-to-I editing, the dsRNA structures are disrupted, reducing the efficiency of back-splicing and circRNA formation [30, 31]. Furthermore, the specific sequence characteristics within the 3′-untranslated region (3′-UTR) typically determine the degradation of both mRNA and circRNA. Depletion of *UPF1* and its associated protein G3BP1 leads to an increase in the steady-state levels of mRNAs and circRNAs that possess highly structured 3′-UTRs [32]. The degradation of circRNA is also associated with specific RNases. Liu et al. [33] discovered that circRNAs were globally degraded by RNase L, a mechanism crucial for activating PKR during early cellular innate immune responses. Park et al. [34] provided evidence regarding the recognition of m6A-modified circRNAs by the YTHDF2 reader protein, which is known to initiate RNA degradation upon m6A modification. They revealed that YTHDF2 facilitated the interaction between RNase P/mitochondrial RNA processing (MRP) and heat-reactive protein 12 (HRSP12) specifically in the presence of m6A modification. As a result, the m6A-modified circRNAs were cleaved by intracellular nucleases, leading to their degradation. Argonaute2 (Ago2) is an endonuclease that carries out its function by relying on endogenous RNA guidance, particularly through miRNAs. *MiR-671* can bind to *circCDR1as* via base pairing and guides the Ago2-dependent degradation of *circCDR1as* [35]. Additionally, GW182 is implicated in the regulation of circRNA degradation through a mechanism that operates independently of Ago2-slicer or P-body. The Mid-domain of GW182 is suggested to play a critical role in the degradation process of circRNA [36].

## Molecular mechanisms of circRNA function

CircRNAs play a crucial role in the initiation and progression of various diseases, including tumors. Diverse molecular mechanisms contribute to their functional roles, such as acting as miRNA sponges, interacting with proteins and mRNAs, regulating the transcriptional processes of host genes, and being translated into functional peptides [37–39]. Many circRNAs can adsorb different types and quantities of miRNAs, form binding competition with mRNA-miRNA, and indirectly increase the expression of proteins by negatively regulating miRNAs [40]. This phenomenon is commonly known as the sponge effect, and the circRNAs involved are referred to as competitive endogenous RNAs (ceRNAs) [41]. For instance, *ciRS-7*, a highly expressed circRNA in both human and mouse brains, contains over 70 selectively conserved miRNA binding sites and forms a strong association with Ago2 in a *miR-7*-dependent manner. *CiRS-7* effectively inhibits the activity of *miR-7*, leading to elevated levels of *miR-7* target genes, providing evidence that *ciRS-7* functions as a sponge for *miR-7* [42]. CircRNAs can also directly bind to proteins and regulate their translocation or function. They can also enhance or disrupt the interaction between two proteins, thereby exerting specific functions [43]. For instance, *circFOXO3* interacts with the anti-senescent protein ID1 and the transcription factor E2F1, as well as the anti-stress proteins FAK and HIF1α, thereby affecting their subcellular localization. The interaction with *circFOXO3* hinders the proper translocation of these proteins, leading to a disruption in their anti-senescent activities [44]. Similarly, another circular RNA *circCCNB1* can interact with both CCNB1 and CDK1, disrupting their interaction by forming a large ternary complex. This disruption restricts their nuclear translocation, consequently impairing the functionality of CCNB1 and resulting in inhibition of tumor growth [45]. Additionally, certain circRNAs can regulate transcription by interacting with RNA polymerase II as well as other transcriptional regulatory factors. For example, *circACTN4* recruits Y-box binding protein 1 (YBX1) to initiate Frizzled-7 (FZD7) transcription [46]. Certain circRNAs are found to play a significant role in the regulation of mRNA stability. For example, *circFIRRE* has the ability to bind to heterogeneous nuclear ribonucleoprotein C (HNRNPC) protein. This interaction serves to modulate the stability of *GLI2* mRNA, thereby promoting the progression of esophageal squamous cell carcinoma [47]. *CircCAMSAP1* exerts a promoting effect on the progression of NPC by enhancing the stability of *SERPINH1*. This is achieved through its binding to the 3’-UTR of *SERPINH1* [48]. *CircBRD7*, a circular RNA derived from its host gene, is found to promote the transcriptional activation of its host gene *BRD7*, thereby attenuating tumor growth and metastasis in NPC [49]. Normally, ncRNAs do not encode proteins, but circRNAs are the exception. In recent years, a large number of studies revealed that some circRNAs could encode peptides and perform translation. For example, *circZNF609* possesses an open reading frame (ORF) that initiates with the same start codon as its linear transcript and terminates with a stop codon formed during circularization. This circRNA associates with ribonucleoprotein particles and is translated into proteins via splice-dependent and cap-independent mechanisms [50]. CircRNAs, utilizing these mechanisms, act as regulators in tumor cells, influencing crucial aspects such as proliferation, apoptosis, invasion, metastasis, angiogenesis, stemness, chemotherapy resistance, and others. They can exert either promoting or inhibitory effects on these processes, thereby modulating tumor progression (Fig. 1).

## The functions of circRNAs in NPC

In recent years, increasing evidence suggests that circRNAs regulate various aspects of NPC progression. Their functions include promoting or inhibiting proliferation, apoptosis, migration, invasion, metastasis, metabolism, angiogenesis and so on by acting as miRNA sponges, interacting with proteins and mRNAs. (Tables 1–3).

**Table 1:** The functions of circRNAs acting as miRNA sponges in NPC.

CircRNA	Expression	Function	Sponge target/miRNA target	Ref
*circCTDP1*	Up	Proliferation(+) Apoptosis(−) Migration(+) Invasion(+)	*miR-320b/HOXA10*	[51]
*circ_0000523*	Up	Proliferation(+)	*miR-1184/COL1A1*	[52]
*circHIPK3*	Up	Proliferation(+) Migration(+) Invasion (+) Metastasis(+)	*miR-4288/ELF3*	[53]
*circSOX9*	Up	Proliferation(+) Migration(+) Invasion (+) Metastasis(+)	*miR-485-3p/SOX9*	[74]
*circZNF609*	Up	Proliferation(+) Migration(+) Invasion(+)	*miR-150-5p/Sp1*	[54]
*circZNF609*	Up	Proliferation(+) Migration(+) Invasion(+) Glycolysis(+)	*miR-338-3p/HRAS*	[78]
*circZNF609*	Up	Proliferation(+) Migration(+) Angiogenesis(+)	*miR-145/STMN1*	[79]
*circ_0081534*	Up	Proliferation(+) Invasion (+)	*miR-5085p/FN1*	[55]
*circTGFBR2*	Down	Proliferation(-) Apoptosis(+) Migration(-) Invasion(-)	*miR-107/TGFBR2*	[60]
*circITCH*	Down	Proliferation(-) Migration(-) Invasion(-) Metastasis(-)	*miR-214/PTEN*	[61]
*circ_0046263*	Up	Proliferation(+) Migration(+) Invasion(+)	*miR-133a-5p/IGFBP3*	[57]
*circCRIM1*	Up	Migration(+) Invasion(+) Metastasis(+)	*miR-422a/FOXQ1*	[68]
*circCRIM1*	Up	Proliferation(+) Migration(+) Invasion(+)	*miR-34c-5p/FOSL1*	[56]
*circTRAF3*	Up	Proliferation(+) Apoptosis(−) Invasion(+) Metastasis(+)	*miR-203a-3p/AKT3*	[64]
*circ_0000215*	Up	Proliferation(+) Migration(+) Invasion(+) Metastasis(+)	*miR-512-5p/PIK3R1*	[59]
*circSETD3*	Up	Invasion(+) Migration(+)	*miR-615-5p and miR-1538/MAPRE1*	[67]
*circMAN1A2*	Up	Proliferation(+) Migration(+) Invasion(+) Metastasis(+)	*miR-135a-3p/UBR5*	[73]
*circNOTCH1*	Up	Proliferation(+) Migration(+) Invasion(+)	*miR-34c-5p/c-Myc*	[58]
*circ_0004788*	Up	Proliferation(+) Apoptosis(−) Migration(+) Invasion(+) Angiogenesis(+)	*miR-515-5p/FGF2*	[62]
*circ_0008450*	Up	Proliferation(+) Apoptosis(−) Migration(+) Invasion (+)	*miR-577/CXCL9*	[63]
*circFIP1L1*	Up (after radiation)	Proliferation(-) Apoptosis(+)	*miR-1253/EIF4A3*	[66]
*circFIP1L1*	Not mentioned	Proliferation(-) Angiogenesis(-)	*miR-125a-5p/VEGFA*	[80]
*circCDR1as*	Up	Proliferation(+) Glycolysis(+)	*miR-7-5p/E2F3*	[77]
*circ_0028007*	Up	Proliferation(+) Apoptosis(−) Migration(+) Invasion(+) Angiogenesis(+)	*miR-656-3p/ELF2*	[65]
*circPARD3*	Up	Stemness(+)	*miR-579-3p/SIRT1*	[82]
*circRPMS1*	Up	Proliferation(+) Apoptosis(−) Invasion (+)	*miR203, miR-31 and miR-451*	[85]

+ promote, - inhibit

**Table 2:** The functions of circRNAs interacting with proteins in NPC.

CircRNA	Expression	Function	Interacting protein	Ref
*circRILPL1*	Up	Proliferation(+) Migration(+) Invasion(+) Metastasis(+)	ROCK1 and IPO7	[13]
*circPVT1*	Up	Migration(+) Invasion(+) Metastasis(+)	β-TrCP	[69]
*circIPO7*	Up	Migration(+) Invasion(+) Metastasis(+)	YBX1	[70]
*circCCNB1*	Down	Migration(-) Invasion(-)	NF90	[75]
*circWDR37*	Up (in highly metastatic cell)	Proliferation(+) Migration(+) Invasion(+) Metastasis(+)	PKR	[72]
*circBART2.2*	Up	Immune Escape(+)	RIG-I	[86]

+ promote, - inhibit

**Table 3:** The functions of circRNAs interacting with mRNAs in NPC.

CircRNA	Expression	Function	Interacting mRNA	Ref
*circCAMSAP1*	Up	Proliferation(+) Migration(+) Invasion(+) Metastasis(+)	*SERPINH1*	[48]
*circCCNB1*	Down	Migration(-) Invasion(-)	*TJP1*	[75]
*circARHGAP12*	Up	Migration(+) Invasion(+) Metastasis(+)	*EZR*	[71]
*circRNF13*	Down	Proliferation(-) Migration(-) Invasion(-) Metastasis(-) Glycolysis(-)	*SUMO2*	[76]

+ promote, - inhibit

## Regulation of proliferation

Evidence has revealed that dysregulated expression patterns of circRNAs are intricately linked to the abnormal proliferation of NPC cells and disruption of cell cycle regulation. Many circRNAs are found to promote NPC cell proliferation. For instance, the aforementioned *circCAMSAP1* can enhance NPC progression by stabilizing *SERPINH1* expression through binding to the 3’-UTR of *SERPINH1*. SERPINH1 inhibits the ubiquitination degradation of c-Myc, thereby promoting its accumulation in the nucleus. Once inside the nucleus, c-Myc facilitates the transcription of the *CAMSAP1* gene and the expression of *SRSF10*. Importantly, c-Myc and SRSF10 participate in the regulation of *circCAMSAP1*, promoting *CAMSAP1* pre-mRNA transcription and back-splicing. This leads to increased *circCAMSAP1* production, establishing a positive feedback loop that drives NPC cell proliferation, migration, invasion and metastasis [48]. *CircRILPL1* is significantly upregulated in NPC. It inhibits the LATS1-YAP kinase pathway by binding to and activating ROCK1, leading to reduced YAP phosphorylation, and facilitating YAP translocation from the cytoplasm to the nucleus by interacting with IPO7. Inside the nucleus, YAP promotes the transcription of *CAPN2* and *PXN* related to cytoskeleton remodeling. By modulating these processes, *circRILPL1* drives NPC cell proliferation, invasion, migration and metastasis [13]. Inhibition of *circCTDP1* leads to a decrease in the proliferative, migration, and invasion capacity of NPC cell lines 6-10B and SUNE2. Mechanistically, *circCTDP1* functions as a *miR-320b* sponge, sequestering *miR-320b* and preventing it from targeting *HOXA10*. *CircCTDP1* promotes NPC progression by modulating the *miR-320b/HOXA10/TGFβ2* pathway [51]. *Circ_0000523* promotes the proliferation of NPC cells and enhances cell cycle progression by targeting the *miR-1184/COL1A1/*PI3K/Akt signaling pathway [52]. In vitro studies showed that the knockdown of *circHIPK3* suppressed NPC cell proliferation, migration, and invasion. In vivo experiments demonstrated that depletion of *circHIPK3* significantly inhibits tumor growth and metastasis. Mechanistically, *circHIPK3* acts as a competing endogenous RNA for *miR-4288*, thereby sequestering *miR-4288* and preventing it from targeting *ELF3* [53]. *CircZNF609* can compete with *miR-150-5p*, resulting in the upregulation of Sp1 and promoting the proliferation, migration and invasion of NPC cells [54]. *Hsa_circ_0081534* enhances the proliferative and invasive capabilities of NPC cells by upregulating FN1 through the sponging of *miR-508-5p* [55]. *CircCRIM1* promotes NPC progression via the *miR-34c-5p/FOSL1* axis. Silencing *circCRIM1* significantly inhibited NPC cells proliferation, migration and invasion [56]. Knockdown of *circ_0046263* inhibits NPC cell proliferation, invasion, and migration, while its overexpression produces the opposite effects. Mechanistically, *circ_0046263* functions as a miRNA sponge by sequestering *miR-133a-5p*, consequently upregulating the expression of its downstream target *IGFBP3* [57]. *CircNOTCH1* exhibits high expression levels in NPC tissues and cells. Silencing *circNOTCH1* leads to the suppression of NPC cell proliferation, invasion, and migration. In terms of mechanism, c-Myc activates *circNOTCH1* by binding to the *NOTCH1* promoter. Interestingly, *circNOTCH1* serves as a competitive endogenous RNA, regulating c-Myc expression by sequestering *miR-34c-5p* [58]. Additionally, *circ_0000215* is overexpressed and exerts oncogenic effects in NPC by functioning as a molecular sponge to suppress the expression of *miR-512-5p*, leading to increased expression of PIK3R1 in NPC cells. Knockdown of *circ_0000215* impedes the growth, migration, invasion and metastasis of NPC cells in vitro and in vivo [59].

Indeed, there are several circRNAs have been found to inhibit the proliferation of NPC cells. A noticeable downregulation of *circTGFBR2* expression is observed in NPC tissue specimens. Experimental data from both in vivo and in vitro studies indicate that *circTGFBR2* plays an inhibitory role in controlling the proliferation of NPC cells. Specifically, *circTGFBR2* directly interacts with *miR-107*, thereby exerting regulatory influence over the expression of *TGFBR2* [60]. NPC cells exhibit a concomitant downregulation of *circITCH* expression. Notably, the expression of *circITCH* is inversely correlated with TNM stage, clinical stage, and lymphatic metastasis in NPC. Overexpression of *circITCH* significantly inhibits NPC cell proliferation, migration, and invasion. This functional impact of *circITCH* has been attributed to its role as a ceRNA, acting as a sponge for *miR-214* and thereby preventing it from targeting *PTEN* [61]. *CircBRD7* plays a pivotal role in the transcriptional activation and expression of its host gene *BRD7*. This is accomplished through the enhancement of histone 3 lysine 27 acetylation (H3K27ac) enrichment within the promoter region of *BRD7*. Consequently, a positive feedback loop is established between *circBRD7* and *BRD7*, exerting inhibitory effects on the cell proliferation, migration, invasion, and metastasis of NPC [49].

## Regulation of apoptosis

Several circRNAs have been found to inhibit apoptosis in NPC. The knockdown of *circCTDP1* results in a significant increase in apoptosis in NPC cell lines SUNE2 and 6-10B, while co-transfection with a miR-320b inhibitor reduces the apoptotic rate [51]. *Circ_0004788* is found to be overexpressed in NPC, and its knockdown significantly reduced cell proliferation, angiogenesis, migration and invasion while promoting apoptosis in NPC cells. This effect is achieved by targeting *miR-515-5p* to regulate the expression of *FGF2* [62]. *Circ_0008450* reduces the inhibitory effect of *miR-577* on *CXCL9*, thereby promoting the oncogenic functions in NPC. Silencing *circ_0008450* leads to the inhibition of cell proliferation, migration and invasion, and an increase in apoptotic cell population in NPC [63]. Elevated levels of *circTRAF3* are observed in patients with NPC who exhibit metastasis. Knockdown of *circTRAF3* suppresses proliferation and invasion, and induces apoptosis in NPC cells. Mechanistically, *circTRAF3* functions as an oncogene by antagonizing the inhibitory effect of *miR-203a-3p* on *AKT3* through sequestration of *miR-203a-3p* in NPC [64]. Moreover, the silencing of *circ_0028007* exerts suppressive effects on cell growth, migration, invasion, and angiogenesis, while promoting apoptosis in NPC cell lines SUNE-1 and 5-8 F. Knockdown of *circ_0028007* enhances apoptosis in SUNE-1 and 5-8 F cells, and this effect is attenuated when *miR-656-3p* is inhibited [65].

There have also been reports of circRNAs that promote apoptosis. Inhibiting *circTGFBR2* leads to a substantial suppression of cellular apoptosis, and this suppressive effect is alleviated by the administration of a *miR-107* inhibitor [60]. *CircBRD7* exhibits a promote effect on inducing apoptosis in NPC cells. Overexpression of *circBRD7* results in the upregulation of p21 expression while downregulating CDK4 expression. Additionally, it induces the expression of the apoptosis marker c-PARP [49]. *CircFIP1L1* is found to exert its regulatory effects by acting as a direct inhibitory binding partner for *miR-1253*. The target gene of *miR-1253* is identified as *EIF4A3*. Through the *miR-1253/EIF4A3* axis, *circFIP1L1* plays a role in regulating NPC cell proliferation, apoptosis, and radiosensitivity. Furthermore, EIF4A3 is observed to bind to *FIP1L1* mRNA transcripts, leading to the formation of *circFIP1L1* and the stabilization of *PTEN* mRNA. Overexpressing *circFIP1L1* and silencing *miR-1253* results in the suppression of NPC cell proliferation, promotion of NPC cell apoptosis, and enhanced radiosensitivity of NPC cells [66].

## Regulation of migration, invasion and metastasis

Numerous studies have provided evidence supporting the notion that circRNAs play a promoting role in cell migration, invasion, and metastasis. Among mentioned above that promote cell proliferation, the majority of circRNAs, including *circCAMSAP1*, *circRILPL1*, *circCTDP1*, *circHIPK3*, *circSOX9*, *hsa_circ_0081534*, *circ_0046263*, *circCRIM1*, *circNOTCH1*, *circ_0000215*, *circ_0004788*, *circ_0008450* and *circTRAF3*, are also reported to facilitate cell migration, invasion or metastasis. Furthermore, a study demonstrated that *circSETD3* in NPC cells acted as a ceRNA, competitively adsorbing to *miR-615-5p* and *miR-1538*. This interaction attenuated the suppressive effects of the miRNAs on *MAPRE1* mRNA, resulting in increased MAPRE1 expression. Elevated levels of MAPRE1 subsequently inhibited α-tubulin acetylation, promoted microtubules dynamic assembly, and enhanced the invasion and migration capabilities of NPC cells [67]. *CircCRIM1* is discovered to be upregulated in highly metastatic NPC cells and NPC tissues with distant metastasis. Overexpression of *CircCRIM1* has been observed to enhance NPC cell metastasis and promote EMT. The mechanism underlying this effect involves *circCRIM1* competitively binding to *miR-422a*, thereby preventing *miR-422a* from suppressing its target gene *FOXQ1* [68]. Mo Y et al. [69] revealed that *circPVT1* exerted an inhibitory effect on the ubiquitin-mediated degradation of c-Myc by forming a binding interaction with β-TrCP. This binding event disrupted the association between the ubiquitin E3 ligase β-TrCP and its substrate c-Myc. In addition, c-Myc and SRSF1 were involved in the regulation of *circPVT1* and promoting *PVT1* pre-mRNA transcription and back-splicing, which led to an increase in the production of *circPVT1*, establishing a positive feedback loop that drive the metastasis of NPC cells. As a result, this molecular interaction led to the remodeling of the cytoskeleton and the modulation of cell adhesion, ultimately promoting the migration, invasion and metastasis of NPC cells. *CircIPO7* exhibits significant overexpression in NPC patients with distant metastasis and promotes NPC cell migration, invasion, and cisplatin resistance in vitro. It interacts with cytoplasmic YBX1, resulting in the phosphorylation of YBX1 at serine 102 by AKT kinase. This event promotes the nuclear translocation of YBX1, thereby activating the transcription of *FGFR1*, *TNC*, and *NTRK1*. As a consequence, *circIPO7* facilitates the migration, invasion and metastasis of NPC [70]. Fan C et al. [71] found that *circARHGAP12* expression was markedly upregulated in both NPC tissues and cell lines, and was associated with the promotion of NPC cell migration and invasion. *CircARHGAP12* was observed to directly bind to the 3′-UTR of *EZR* mRNA, enhancing its stability. EZR protein formed a complex with TPM3 and RhoA, facilitating NPC cell invasion and metastasis. Li Q et al. [72] uncovered a mechanism of *circWDR37* activated PKR in senescence-driven metastasis. *CircWDR37* interacts with and forms dimers with double-stranded RNA-activated protein kinase R (PKR), leading to the initiation of PKR autophosphorylation and activation. The phosphorylated PKR then induces the phosphorylation of I-kappaB kinase beta (IKKβ). This phosphorylated IKKβ subsequently releases RELA from NF-κB inhibitor alpha (IκBα), resulting in the activation of NF-κB. This *circWDR37*-dependent activation of NF-κB stimulates the transcription of *CCND1* and genes associated with the senescence-associated secretory phenotype (SASP), contributing to proliferation, migration, invasion and metastasis of NPC. Knockdown of *circMAN1A2* significantly impedes the proliferation, migration, invasion, and metastasis of NPC. The underlying mechanism involves *circMAN1A2* acting as a sponge for *miR-135a-3p*, sequestering *miR-135a-3p* and preventing its inhibitory effect on *UBR5* [73]. Sun Y et al. [74] revealed that the expression of *circSOX9* was correlated with lymphatic metastasis and distant metastasis in NPC. They found that *circSOX9* acted as a molecular sponge for *miR-485-3p* and prevented it from targeting *SOX9*, promoting the proliferation and invasion of NPC cell lines HONE1 and CNE2.

There is limited literature reporting the inhibitory effects of circRNA on the migration, invasion, or metastasis of NPC. The previously mentioned *circTGFBR2* has also demonstrated its ability to suppress the migration and invasion of NPC cells by sponging *miR-107* [60]. Another study revealed that *circCCNB1* can regulate TJP1, a key regulator of tight junction assembly that coordinates the assembly or dynamics of the cortical cytoskeleton and regulates adhesion function. *CircCCNB1* inhibits NPC migration and invasion by promoting NF90 binding to *TJP1* mRNA and stabilizing it, and enhancing tight connections between tumor cells [75].

## Regulation of metabolism

There is a close relationship between metabolism and cancer. Metabolic pathways undergo significant alterations in tumor cells to meet their specific biological behaviors. *CircRNF13* is found to have stable low-level expression in NPC clinical tissues and NPC cells. Both in vitro and in vivo experiments demonstrate that *circRNF13* inhibits NPC proliferation, migration, invasion and metastasis. Furthermore, *circRNF13* activates the SUMO2 protein by binding to the 3’-UTR of the *SUMO2* gene, thus prolonging the half-life of *SUMO2* mRNA. The increased levels of SUMO2 promotes GLUT1 degradation through SUMOylation and ubiquitination of GLUT1, which regulates the AMPK-mTOR pathway. This ultimately leads to the inhibition of glycolysis, resulting in the suppression of NPC proliferation and metastasis [76]. The expression of *circCDR1as* is significantly upregulated in NPC tissues compared to non-tumor NP tissues, indicating an association with poor prognosis in NPC patients. Additionally, *circCDR1as* is found to upregulate E2F3 expression by binding to *miR-7-5p*, thereby promoting proliferation and glucose metabolism of NPC cells [77]. *CircZNF609* functions as a ceRNA for *miR-338-3p*, thereby regulating *HRAS* expression. Knockdown of *circZNF609* leads to the suppression of cell proliferation, migration, invasion, and glycolysis in NPC through the modulation of the *miR-338-3p/HRAS* axis [78].

## Regulation of angiogenesis

Angiogenesis plays a pivotal role in both physiological and pathological contexts, notably in cancer. In NPC, several studies have reported the regulatory role of circRNAs in angiogenesis. The expression of *circ_0004788* is found to be elevated in NPC, and its depletion results in a significant reduction in angiogenesis in NPC cells [62]. *CircZNF609* functions as a ceRNA to negatively regulate *miR-145* expression. Silencing *circZNF609* results in the suppression of cell proliferation, migration, and angiogenesis in NPC. However, these effects are reversed when knocking down of *miR-145*. *STMN1* is identified as a downstream target of *miR-145*. Overexpression of *miR-145* suppresses cell proliferation, migration, and angiogenesis in NPC, but this effect is abolished by STMN1 overexpression [79]. Zhou T et al. [80] found that *miR-125a-5p* exhibited high expression levels in both NPC tissues and cells. Overexpression of *miR-125a-5p* accelerated the proliferation and angiogenesis of human umbilical vein endothelial cells (HUVECs). Knockdown of *miR-125a-5p* inhibited the expression of vascular endothelial growth factor A (*VEGFA*). Furthermore, exosomal *circFIP1L1* secreted from the NPC cell line HNE1 acted as a sponge for *miR-125a-5p*, thereby inhibiting the *VEGFA* pathway and suppressing angiogenesis in HUVECs. Moreover, the knockdown of *circ_0028007* is found to have suppressive effects on NPC cell angiogenesis in vitro. Mechanistically, *circ_0028007* silencing is found to regulate the AMPK/mTOR pathway in NPC cells. *Circ_0028007* acts as a sponge for *miR-656-3p* and elevates its target gene *ELF2* expression, thereby promoting the malignant behaviors of NPC cells [65].

## Regulation of tumor microenvironment

The tumor microenvironment (TME) exerts a pivotal influence on tumor growth, progression, and therapeutic response. It has been confirmed that circRNAs can modulate the TME in certain cancers. CircRNAs exert their influence on the TME through various mechanisms, including the regulation of intercellular communication, modulation of substances secreted by tumor cells, and control over immune responses. These regulatory effects can enhance tumor growth, facilitate invasion, and enable evasion of immune surveillance, ultimately affecting tumor development and treatment responses. Thus, the role of circRNAs in the TME has emerged as a crucial area of research in the field of oncology. However, currently, there is little research in this field in NPC. In a study conducted by Wang Y et al. [81], it was observed that the ratio of T cells within the TME undergoes changes in recurrent NPC compared to primary NPC. The five differentially expressed circRNAs, including *hsa-circ-0006935*, showed high expression levels in T cells and NPC tissues. Furthermore, the expression of certain circRNAs were found to be higher in CD3+ cells compared to CD3- cells. These findings suggest the potential involvement of circRNAs in the TME during tumor recurrence in NPC. However, further research is needed to explore the precise role and mechanisms of circRNAs in this context.

## Regulation of stemness

Cancer stemness has been acknowledged as the principal driver of cancer metastasis and recurrence. Ai et al. [82] reported that *EBV-miR-BART4* could induce stem-like properties and cisplatin resistance in NPC-SP cells. Mechanistically, exosomes loaded with *circPARD3* promote *EBV-miR-BART4*-induced stemness and cisplatin resistance through regulating the *miR-579-3p/SIRT1/SSRP1* axis. However, further research is needed to using more NPC cell lines and larger sample size. Currently, there is limited research on the regulation of NPC stemness by circRNAs, and more circRNAs with regulatory effects in this field are yet to be discovered.

## Functions of EBV-encoded circRNAs in NPC

EBV is the first human virus discovered to encode miRNAs, and it is causally associated with the development of NPC. Recent studies have revealed that EBV is capable of encoding circRNAs. Researchers conducted RNA sequencing on ribosome-depleted total RNA from some EBV-positive cells, including SNU-719, AGS-EBV, C666-1, and Akata. They identified an EBV-encoded circRNA named *ebv_circ_RPMS1*, which was found in both the cytoplasm and nucleus [83]. In addition, Toptan et al. [84] also observed the presence of EBV-encoded circRNAs, known as *circBARTs*, in all confirmed EBV latency types, including NPC. This finding suggests that *circBARTs* may play a role in viral oncogenesis in EBV-associated tumor. Specifically, EBV-encoded *circRPMS1* has been shown to be frequently upregulated in EBV-positive NPC and its increased expression is associated with poor survival outcomes. *CircRPMS1* has the capacity to interact with multiple tumor-suppressive miRNAs, including *miR-203*, *miR-31*, and *miR-451*. Knockdown of *circRPMS1* inhibited proliferation and invasion, and induced apoptosis in EBV positive NPC cells. These findings suggest that targeting *circRPMS1* could hold promise as a therapeutic strategy for EBV-associated NPC [85]. C*ircBART2.2* is highly expressed in NPC and has been found to significantly upregulate the expression of PD-L1, thereby contributing to immune evasion by NPC cells. The mechanism involves *circBART2.2* binding to the helicase domain of RIG-I, leading to the activation of transcription factors *IRF3* and *NF-κB* [86].

**Fig. 2:**
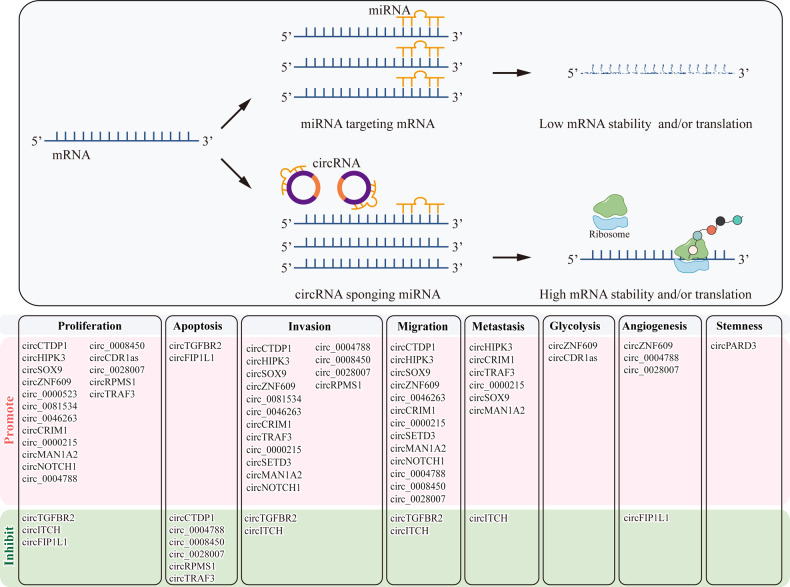
CircRNAs regulate the progression of NPC by functioning as miRNA sponges. By binding to miRNAs, circRNAs can prevent them from targeting downstream mRNA, thereby affecting mRNA stability and/or translation. This mechanism allows circRNAs to regulate various biological behaviors in NPC, including promotion or inhibition of proliferation, invasion, migration, metastasis, apoptosis, angiogenesis, glycolysis, and stemness.

**Fig. 3:**
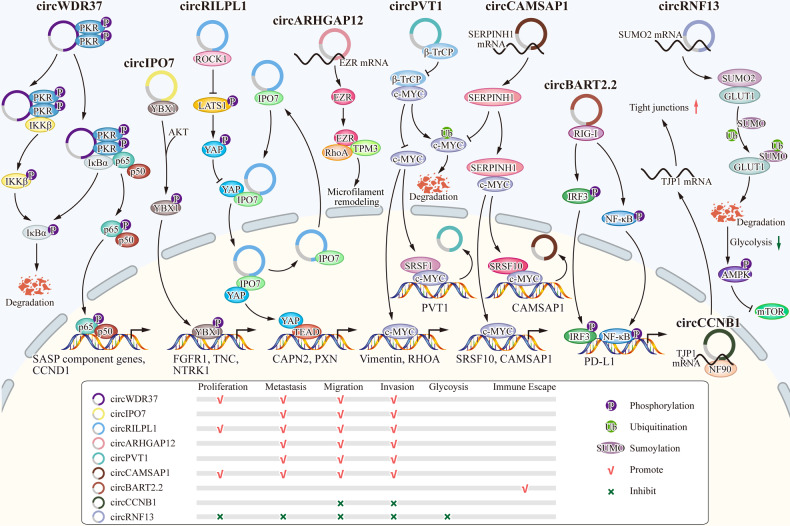
CircRNAs regulate the progression of NPC by interacting with proteins and mRNAs. *CircWDR37*, *circIPO7*, *circRILPL1*, *circPVT1* and *circCCNB1* can bind to proteins, activating them, facilitating transportation, or competitively binding to target proteins, thereby modulating downstream signaling pathways. Additionally, *circARHGAP12*, *circCAMSAP1*, *circRNF13* and *circCCNB1* can regulate the stability and expression of mRNA by binding to mRNA molecules, thereby affecting protein expression and function.

As described above, circRNA plays a variety of functions in the occurrence and development of NPC, and these functions are generated through various mechanisms, such as acting as miRNA sponges (Fig. 2) and interacting with proteins and mRNAs (Fig. 3).

## CircRNAs as potential biomarkers in NPC

Due to the stable characteristics of circRNA, recent studies have investigated the diagnostic and prognostic potential of specific circRNAs in the development and progression of NPC. For instance, the expression of *hsa_circ_0066755* is significantly increased in both the plasma and tissues of NPC patients. Its diagnostic accuracy in tissue and plasma was comparable to that of magnetic resonance imaging (MRI), making it a valuable diagnostic marker for NPC [87]. A study conducted by Fan et al. [88] revealed that the expression of *circMAN1A2* significantly increased in the serum of patients with NPC, oral cancer, thyroid cancer, ovarian cancer, and lung cancer, suggesting its promising clinical diagnostic value as a serum biomarker for multiple malignancies and offering potential insights into early cancer diagnosis. However, further studies are needed to clarify the functions and regulatory mechanisms of *circMAN1A2* in the progression of malignant cancers. Elevated expression of *circRILPL1* in NPC has been associated with unfavorable prognostic outcomes. This indicates its potential as a significant prognostic indicator and a valuable diagnostic biomarker for NPC [13]. Furthermore, a study investigated the prognostic value of *circIPO7* in NPC patients undergoing cisplatin-based chemotherapy. The results revealed that high *circIPO7* expression was linked to unfavorable distant metastasis-free survival (DMFS). Patients with high *circIPO7* expression had significantly worse overall survival (OS), disease-free survival (DFS) and DMFS compared to those with low *circIPO7* expression. These findings suggest that *circIPO7* could serve as a valuable prognostic biomarker for NPC patients receiving cisplatin-based chemotherapy [70]. *CircRNA_0000285* displays a substantial upregulation in both NPC tissues and the serum of NPC patients. A significant correlation is observed between *circRNA_0000285* and several clinical parameters, including tumor size, differentiation, lymph node metastasis, distant metastasis, and TNM stage. *CircRNA_0000285* acts as an independent prognostic factor, influencing the outcome and prognosis of NPC patients. These findings suggest that *circRNA_0000285* holds promise as a novel biomarker for NPC [89]. In addition, *hsa_circRNA_001387* shows a significant correlation with various factors including differentiation, lymph node metastasis, distant metastasis, TNM staging and EBV in NPC patients and demonstrated high accuracy in diagnosing NPC, suggesting its potential as a specific and sensitive diagnostic marker. Moreover, *hsa_circRNA_001387* is also identified as an independent factor for predicting the prognosis of NPC patients [90].

## CircRNAs as potential therapeutic targets in NPC

Several publications have discussed the potential effects of circRNA in the treatment of NPC. For example, *circRNA_000543* shows promise as a potential target for overcoming radiation resistance in NPC. The expression of *circRNA_000543* is found to be significantly higher in radioresistant NPC samples compared to radiosensitive NPC samples. NPC patients with elevated *circRNA_000543* expression show poorer overall survival. The mechanism underlying the sensitization of NPC cells to radiotherapy upon *circRNA_000543* knockdown involves the *circRNA_000543/miR-9/PDGFRB* axis [91]. *CircCRIM1* is upregulated in highly metastatic NPC cells and NPC tissues with distant metastasis. It acts as a competitive sponge for *miR-422a*, counteracting *miR-422a* suppressive effect on *FOXQ1* expression, thereby promoting docetaxel chemoresistance [68]. *CircSETD3* can sequester *miR-147a*, leading to the activation of the Akt/mTOR pathway and promoting cisplatin resistance in NPC [92]. Depletion of *circWDR37* increases sensitivity to chemotherapy-induced senescence and enhances chemotherapeutic efficacy, suggesting its potential to serve as a biomarker for predicting chemotherapy response and as a therapeutic target in NPC [72]. Luo Y et al. [93] discovered that circulating exosomal *circMYC* holds promise as a potential therapeutic target for NPC. They observed a significant elevation of circulating exosomal *circMYC* in NPC patients, which correlated with tumor characteristics and patient outcomes. Functional experiments revealed that overexpression of *circMYC* promoted cell proliferation and reduced radiosensitivity in NPC. These findings highlight the potential of circulating exosomal *circMYC* as a therapeutic target for NPC. *CircRNA_0067717* shows substantial upregulation in paclitaxel-resistant NPC cells and is closely associated with the development of paclitaxel resistance. Notably, in paclitaxel-resistant NPC cells, elevated expression of *circRNA_0067717* promotes the interaction between TRIM41 and p53 proteins, facilitating TRIM41-mediated ubiquitination and subsequent degradation of p53 [94]. In patients with chemotherapy-resistant NPC, there is a prominent upregulation of *circNRIP1* expression. *CircNRIP1* acts as a ceRNA for *miR-515-5p*, effectively sequestering it and preventing its suppressive effect on IL-25 expression. *CircNRIP1/miR-515-5p/IL-25* regulatory axis provides valuable insights into the mechanisms underlying 5-Fu and cisplatin resistance in NPC. Targeting this axis may offer a promising therapeutic strategy for treating NPC and overcoming chemotherapy resistance [95]. Yang et al. [96] established radioresistant models and compared circRNA expression profiles between radioresistant cell line and non-radioresistant cell line using high-throughput microarrays. 1042 upregulated circRNAs and 1558 downregulated circRNAs were identified in radioresistant cells. However, further functional and mechanistic experiments are required to identify specific circRNAs associated with radioresistance and explore their therapeutic value in the treatment of NPC.

The studies aforementioned above provide valuable insights into the potential utilization of circRNAs as therapeutic targets in the treatment of NPC. However, it is important to consider the potential challenges and limitations associated with translating these findings into clinical applications. Novel therapeutic approaches often come with unforeseen side effects and safety concerns, which need to be thoroughly evaluated. The heterogeneous characteristic of NPC and the individual variability in patient responses necessitate personalized treatment strategies for optimal outcomes. Additionally, the development and implementation of new therapies can be costly and may face obstacles in terms of accessibility, particularly in resource-limited settings. Furthermore, the emergence of treatment resistance is a common issue in cancer therapy, and there is a possibility that NPC cells may develop resistance to circRNA-targeted treatments. Therefore, it is crucial to identify reliable biomarkers that can accurately predict treatment response and guide patient selection for these novel therapies. Despite these challenges, the exploration of circRNAs as therapeutic targets in NPC holds promise and warrants further investigation to improve the clinical management of this disease.

## Conclusions

This review provides a comprehensive analysis of the diverse functions of circRNAs in NPC, highlighting their impact on various biological processes and their potential as valuable targets for diagnosis, treatment and prognosis. The intricate involvement of circRNAs in NPC pathogenesis, including their influence on cell proliferation, apoptosis, migration, invasion, metastasis, metabolism, angiogenesis, TME and drug resistance, emphasizes their significance in NPC. The unique characteristics of circRNAs, such as their stability, abundance, and cell-type specific expression patterns, make them promising non-invasive biomarkers for early detection and prognostic assessment of NPC. Additionally, this review sheds light on the specific role of EBV-encoded circRNAs in NPC, expanding our understanding of the contribution of viruses to cancer development. However, the research on circRNA mechanisms in NPC is still incomplete, and the translation function of circRNAs in NPC occurrence and development has not received sufficient attention. Furthermore, most studies lack validation in large-scale clinical cases. Thus, there are challenges ahead, including the need for a deeper understanding of circRNA mechanism and validation in larger and more diverse populations.

We believe that circRNAs have good clinical application prospects in the diagnosis, treatment, and prognosis of NPC. Given the close relationship between EBV and NPC and the high specificity of EBV transcripts in NPC, the detection of EBV-encoded circRNAs, such as *circRPMS1*, may have broad potential for application in the diagnosis and monitoring of NPC. Based on the differences in the function of circRNAs in NPC, personalized medicine markers may need to take circRNAs into account. Monitoring and targeting the expression of oncogenic circRNAs could also be an important way to evaluate the therapeutic effect and adjust the therapeutic strategies. Developing a treatment plan targeting oncogenic circRNA based on individualized circRNA expression profiles in NPC patients may be a feasible intervention strategy in the future. Furthermore, resistance to radiation therapy and chemotherapy represents significant challenges in the management of NPC. Targeting circRNA known to confer resistance to these treatments holds promise to overcome this obstacle and improve the management of NPC. In addition, certain circRNAs are associated with the survival of NPC, indicating their potential prognostic values. However, the current evidence is still very limited, and more circRNAs related to prognosis need to be discovered and studied.

In conclusion, circRNAs represent a frontier in NPC research and have promising clinical applications. The contribution of circRNAs in NPC not only enhances our understanding of the disease’s molecular landscape, but also unveils new avenues for advanced methodologies in diagnosis, prognosis and therapeutic intervention. As research in this field progresses, it is anticipated that circRNA-based interventions will play a significant role in improving outcomes for NPC patients. Although current studies suggest that circRNAs hold great potential as biomarkers and therapeutic targets, their clinical applications in the management of NPC are still at a very preliminary stage. Further clinical studies are needed to validate the feasibility of using circRNAs as markers for diagnosis, treatment, and prognosis of NPC.
